# Assessing the psychological distress and coping strategies among academic staff of a university during COVID-19

**DOI:** 10.4102/hsag.v30i0.2752

**Published:** 2025-03-21

**Authors:** Isaiah Owoeye, Toluwani Agunbiade, Adebanke Agboola, Oluwafemi Sanya, Babatope Adebiyi, Furaha Akimanimpaye

**Affiliations:** 1 School of Nursing, Faculty of Community & Health Sciences, University of the Western Cape, Bellville, South Africa; 2 State Specialist Hospital, Medfestcareng, 48, Ikere Road, Ado-Ekiti, Nigeria; 3 Faculty of Medical & Health sciences, Afe Babalola University, Ado-Ekiti, Nigeria; 4 Department of Computer Science, Faculty of Science, Afe Babalola University, Ado-Ekiti, Nigeria; 5 Centre for Interdisciplinary Studies of Children, Families and Society, Faculty of Community and Health Sciences, University of the Western Cape, Cape Town, South Africa; 6 School of Nursing, Faculty of Community & Health Sciences, University of the Western Cape, Cape Town, South Africa

**Keywords:** COVID-19, psychological distress, coping strategy, academic staff, university

## Abstract

**Background:**

The coronavirus disease 2019 (COVID-19) pandemic has been associated with stress because of its disruption to normal lifestyle. While the resilience of people was challenged, some coping strategies were adopted to maintain balance in the face of the pandemic.

**Aim:**

To assess psychological distress and coping strategies among the academic staff.

**Setting:**

Afe Babalola University located in the Southwest, Nigeria.

**Methods:**

A descriptive-cross-sectional design was used on the population of 512 academics where a sample size of 248 was drawn using Taro Yamane with a 10% non-response rate. The instruments used were a modified Kessler Psychological Distress Scale (K10) and an adapted COPE inventory for coping strategies. The scale reliability of K10 was 0.866 while that of coping strategy was 0.610. Data analysis was performed using Statistical Package for Social Sciences (SPSS) version 28. The results were presented in simple percentages, means and standard deviations.

**Results:**

Most respondents had severe psychological distress (185, 98.9%) with the most rated report ‘Feel worthless’ 4.8 ± 0.59. The most rated coping strategy was ‘I try to lose myself for a while by drinking alcohol or taking drugs’3.8 ± 0.60 with overall coping scale mean, 2.3 ± 1.02.

**Conclusion:**

There was severe distress and substance used among academics. The study recommends teaching on effective coping styles and institution probable preparation for future pandemic.

**Contribution:**

The study provides insight into the psychological state of the academic staff during the COVID-19 pandemic and unveils the adaptive strategies used. The results of the study are useful for the development of appropriate coping skills for the staff.

## Introduction

Psychological distress is a situation of mental fatigue resulting in the person’s inability to adapt to the demands presented by the environment (Watson 2023). The World Health Organization (WHO) referred to psychological distress as an individual’s perception of emotional discomfort, which comes with an alteration in individual well-being. According to the American Psychological Association (APA), psychological distress is a cluster of emotional disorders involving fear and depression as corresponding responses (Belay et al. 2021). The APA manual stressed the inability to cope with usual functions as an issue in psychological distress. This implies that an alteration in daily function is a hallmark of an individual experiencing psychological distress. The range of emotions presented by a psychologically distressed individual is measured in overt and covert behaviours as self-reported responses such as; hopelessness, depression, restlessness, nervousness, sadness, worthlessness, among others (Every-Palmer et al. 2020).

Contextually, the academic staff members are referred to as the teaching personnel of the university. These set of people are primarily employed to teach their various areas of specialties across the departments and colleges of the university. The disruptions on account of the coronavirus disease 2019 (COVID-19) affected the academic staff as Nigeria announced the closure of all academic institutions (Ajide, Ibrahim & Alimi 2020) and the National University Commission shut down universities on 23 March 2020. This happened following the WHO designation of COVID-19 as a pandemic condition on 12 March 2020 (Kontoangelos, Economou & Papageorgiou 2020). The academic interruption caused a plethora of psychological distress among the academic staff (Every-Palmer et al. 2020). Some of the behavioural outcomes of the distress were worthlessness, hopelessness and depression, among others. High levels of psychological distress were recorded among academic staff and its effects were severe depression, sleeplessness and anxiety (Watson 2023). The height of the psychological distress among the academic staff was between the months of April and May 2020 (Tanifuji et al. 2023).

The COVID-19 period resulted in a panic situation that made humans uncomfortable all over the world (Kontoangelos et al. 2020). Coronavirus disease 2019 introduced anxiety into people’s consciousness because of the unknown prognosis of its outcome (Kontoangelos et al. 2020). The anxiety emanating from COVID-19 news was associated with discrimination and death (Salari et al. 2020). The academic staff were caught up in this range of anxiety because they are part of the global system (Stapleton, Garby & Sabot 2020). The regular academic activities were moved online, thereby disrupting the normal interaction as lockdown and social-distancing further compounded the worries (Every-Palmer et al. 2020). In addition, the stress of adaptation to virtual teaching during the pandemic was also recorded among the academic staff (Lassri 2023). The academic staff found the transition to virtual teaching a challenging task (Leal Filho et al. 2021). The quick adoption of a non-traditional virtual lecture by institutions imposed stress on the academic staff because they were not mentally ready for it (Ebohon et al. 2021), as learning to use an alternative such as virtual learning was burdensome (Leal Filho et al. 2021). As these myriads of stressors unfolded, the news about the death and hospitalisation of individuals across the world worsened the palpable anxiety (Li et al. 2021). There were also reported cases of retrenchment of academic staff in schools (Yunusa et al. 2021). This scenario informed the need for coping strategies to mitigate the psychological distress among the academic staff.

Coping strategies are the behaviours that are employed to deal with the stressors originating from within or outside the human environment (Algorani & Gupta 2023). Some of the coping methods such as meditation, playing music and exercise are social skill training used to mitigate the distress (Monfared et al. 2021). In a study that assessed the coping strategy used by the faculty members, the use of substance, avoidance, emotional venting, denial and mental disengagement were recorded as coping mechanisms employed (Mallhi et al. 2023). The distress could be addressed by tackling the problem, using positive emotion, cognitive appraisal and appropriate social support (Algorani & Gupta 2023). The impact of institutional support in mitigating psychological distress among the academic staff cannot be over-emphasised. Institutions provided training and other logistics needed for the academic staff to access and facilitate online teaching (Ebohon et al. 2021), and the support provides a buffer action against stress and gives the staff a sense of mental stability (Liu & Aungsuroch 2018).

There were available studies that focussed on psychological distress among the academic staff during the COVID-19 pandemic (Akour et al. 2020; Hutchison et al. 2022; Saravanan et al. 2020). Studies were also carried out to investigate the impact of COVID-19 on the academic staff (Akour et al. 2020; Ebohon et al. 2021; Yunusa et al. 2021). There were also few research reports, which are foreign-based studies, that investigated both psychological distress and coping strategies among the academic staff (Gustems-Carnicer et al. 2020; Mallhi et al. 2023; Shen & Slater 2021; Stapleton et al. 2020). The researcher, however, found that no study has been conducted on psychological distress and coping strategies among Nigerian academic staff during the COVID-19 pandemic. This gave an impetus for the investigation to generate data and for probable preparation to manage the future pandemic situation that could challenge the psychological states of the academic staff.

### Objectives of the study

1. To assess psychological distress among the academic staff of the university.
2. To identify coping strategies used by the academic staff of the university.

### Hypothesis

There will be no significant difference between academic staff demographics and psychological distress.

## Research methods and design

### Study design

This is a descriptive-cross sectional design conducted at Afe Babalola University, Ado-Ekiti, Nigeria.

### Population and sample

The population for this study was academic staff members of the university, which comprises males and females across all department and/or colleges of the institution. The staff were from different geo-political zones of the country and have years of teaching experience. The total number of the academic staff was 512. The sample size for the study was 248 using Taro Yamane sample size formula and 10% non-response rate.

### Instrument

The instruments used for data collection were standardised tools; Kessler Psychological Distress (K10) Scale for psychological distress and an adapted copy of Brief Coping Orientation for Problem Experiences (COPE) to measure coping strategies. The statements in the coping strategies are classified under; substance use, behavioural disengagement, focus on and venting of emotions, mental disengagement, planning, active coping, and positive reinterpretation and growth. Researchers have adapted the instrument to suit studies over time (Brambila-Tapia et al. 2023). The scale reliability of psychological distress was 0.866, while that of coping strategy was 0.610. Data were collected between August and September 2020, following the school resumption from the lockdown. Strict adherence to COVID-19 rules was followed and data collection lasted for 3 weeks.

### Data analysis

The data were cleaned and analysed using Statistical Package for Social Sciences (SPSS) version 28. Descriptive and inferential statistics were used for the data. Kessler-10 (K-10) comprises 10 questions ranging from 1 to 5, from ‘None of the time’ to ‘All of the time’, respectively. The score of these questions was summed up to 50 with the minimum being 10 and maximum 50. The following ranges were denoted as the level of psychological distress: 10–19 for ‘Likely to be well’, 20–24 ‘likely to have a mild disorder’, 25–29 ‘likely to have a moderate disorder’ and w30–50 ‘likely to have a severe disorder’. A statement from the K-10 was removed to improve the reliability index, and analysis was done according to the instructions on the usage of the instrument. The data were presented using mean and standard deviation, while chi-square and Kruskal-Wallis tests were used to analyse the relationship between the demographics and psychological distress. Multiple regression analysis was also performed to predict the impact of psychological distress on coping strategies.

### Ethical consideration

Ethical clearance was obtained from the Research and Ethics Committee of Afe Babalola University, Ado-Ekiti, Nigeria. AB/EC/20/07/143. A written consent was obtained from the respondents, and the information given was made anonymous and strictly confidential with no document having the identity of the respondents.

## Results

There were 187 respondents in this study, and the response rate was 75.4%. The result showed that 83 (44.4%) respondents were between the ages 30–39 years. Over three-fifths of the respondents (116, 62.0%) were male. Over one-quarter of the respondents (59, 31.6%) were from the College of Engineering. Over three-quarters of the respondents (148, 79.1%) had 1–10 years of teaching experience (Table 1).

**TABLE 1:** Socio-demographic variable (*N* = 187).

Items	Outcomes	
	*n*	%
**Age (years)**		
20–29	11	5.9
30–39	83	44.4
40–49	67	35.8
50–59	18	9.6
60–69	8	4.3
**Gender**		
Male	116	62.0
Female	71	38.0
**Colleges**		
Engineering	59	31.6
CMHS	56	29.9
Science	36	19.3
SMS	28	15.0
Law	8	4.3
**Years of teaching experience**		
1–10	148	79.1
11–20	33	17.6
21–30	4	2.1
31 and above	2	1.1

CMHS, College of Medicine and Health Sciences; SMS, Social and Management Sciences.

Psychological distress was measured using nine statements from Kessler-10. The highest reported statement was ‘Feel worthless’ 4.8 ± 0.59, while the least rated statement was ‘Everything was an effort’ 4.3 ± 0.78. The total means of the scale was 4.6 ± 0.71 and the level of psychological distress among the respondents indicated a likelihood of severe stress (185, 98.9%) (Table 2).

**TABLE 2:** Psychological distress of the academic staff (*N* = 187).

Statements	Mean	s.d.	Frequency	%
Feel worthless	4.8	0.59	-	-
Hopeless	4.7	0.57	-	-
So nervous that nothing could calm you down	4.6	0.78	-	-
So sad that nothing could cheer you up	4.6	0.70	-	-
Depress	4.6	0.69	-	-
Restless you could not sit still	4.6	0.79	-	-
Restless or fidgety	4.5	0.77	-	-
Feel nervous	4.4	0.76	-	-
Everything was an effort	4.3	0.78	-	-
**Level of psychological distress**				
Likely to have a moderate stress	-	-	1	0.5
Likely to have a severe stress	-	-	185	98.9

s.d., standard deviation.

The coping strategy of the respondents was measured using eight statements from the COPE Inventory instrument. The most-rated statement was ‘I try to lose myself for a while by drinking alcohol or taking drugs’ 3.8 ± 0.60 while the least-rated statement was ‘I look for something good in what is happening’ 1.9 ± 1.16. The overall statements revealed that 2.3 ± 1.02 (Table 3).

**TABLE 3:** Coping strategies used by the academics (*N* = 187).

Statements	Category	Mean	s.d.
I try to lose myself for a while by drinking alcohol or taking drugs.	Substance abuse	3.8	0.60
I admit to myself that I can’t deal with it, and quit trying.	Behavioural disengagement	3.6	0.70
I get upset, and am really aware of it.	Focus on and venting of emotions	3.3	0.70
I turn to work or other substitute activities to take my mind off things.	Mental disengagement	3.0	0.97
I think hard about what steps to take	Planning	2.6	1.11
I do what has to be done, one step at a time.	Active coping	2.1	1.16
I look for something good in what is happening.	Positive reinterpretation and growth	1.9	1.16

Note: Mean 2.9.

s.d. standard deviation.

There were significant differences between age, colleges and psychological distress (*p* < 0.05). Pairwise comparison of age showed differences exist between ‘20–29 years and 30–39 years’ and ‘40–49 years and 30–39 years’. In colleges, the differences were between ‘Engineering and Science’, ‘Engineering and Medicine/Health’, ‘Engineering and Law’, Social Management sciences and Medicine/Health’ and ‘Social management science and Law’ (Table 4).

**TABLE 4:** Demographics and psychological distress (*N* = 187).

Variables	Kessler scale (mean, 41.03)	Test	*p*
**Age (years)**			
20–29	67.50	*K* = 9.5	0.049*
30–39	105.87	-	-
40–49	84.91	-	-
50–59	85.14	-	-
60–69	103.31	-	-
**Gender**			
Male	89.89	χ^2^ = 1.3	0.197
Female	100.71	-	-
**College**			
Medicine & Health	116.47	-	-
Social & Management	81.00	*K* = 29	0.001*
Sciences	102.44	-	-
Law	129.13	-	-
Engineering	68.92	-	-
**Year(s) of experience**			
1–10	91.06	*K* = 3.8	0.292
11–20	107.80	-	-
21–30	74.75	-	-
31 and above	122.00	-	-

* , Significant *P* < 0.05.

Multiple regression analysis was done. The overall fit of the model was significant, as illustrated with F- statistic of 16.20 with a *p*-value less than 0.05 (*F* (7, 179) =16.20, *p* < 0.05). This implies that the model explains a significant portion of the variance in psychological scores. The adjusted *R*^2^ value of 0.364 further denoted that the model accounted for approximately 36% of the final psychological scores variability, showing that the included predictors, a unit increase in substance abuse increased psychological distress by 1.426 units (95% confidence interval [CI]: −0.46 to 1.29) and a unit increase in focus on and venting of emotions increased psychological distress by 1.984. However, a unit increase in planning and positive reinterpretation decreased psychological distress by −0.085 (95% CI: −0.46 to 0.74) and −0.976 (95% CI: −0.61 to 0.46), respectively (Table 5).

**TABLE 5:** Coping strategies and psychological distress (*N* = 187).

Coping category	B	s.e	Beta	*t*	*p*	95% CI
Substance abuse	1.426	0.319	0.278	4.465	0.001*	−0.46 to 1.29
Behavioural disengagement	1.751	0.464	0.246	3.778	0.001*	0.75–2.40
Focus on and venting of emotions	1.984	0.462	0.292	4.295	0.001*	0.97–2.61
Mental disengagement	1.460	0.492	0.056	0.936	0.351	0.72–1.85
Planning	−0.085	0.301	−0.020	−0.282	0.778	−0.46 to 0.74
Active coping	0.158	0.338	0.035	0.468	0.640	−1.46 to 0.30
Positive reinterpretation and growth	−0.976	0.325	−0.226	−3.000	0.003*	−0.61 to 0.46

CI, confidence interval; s.e., standard error.

* , Significant *p* < 0.05.

## Discussion

In this present study, 83 (44.4%) respondents were between the ages of 30–39 years. This is similar to a study where the mean age of the students was 36.14 years (Monfared et al. 2021). The study showed that over three-fifths (116, 62.0%) were male. This is in contrast with a similar study where 147 (74%) accounted for the number of female teachers. Another finding revealed that (148, 79.1%) had between 1 and 10 years of experience. In a documented study carried out among high school teachers in Australia, the mean years of teaching experience was 9.4 (Stapleton et al. 2020).

### Psychological distress

The majority of the respondents 185 (98.9%) had a severe level of distress. The highest reported statement on distress scale was ‘Feel worthless’ 4.8 ± 0.59. The grand mean of the scale was 4.6 ± 0.71, and the level of psychological distress among the respondents indicated a likelihood of severe stress (185, 98.9%). This is not in accordance with another study where the respondents reported an overall severe distress of 0.9% (Li et al. 2021). In another study by Saravanan et al. (2021), in students at a university, the mental distress was 51% and the study associated it with a lack of physical interaction, restrictions solely to the individuals’ house and movement of lectures from conventional four-walls of the school building to digital space. According to a documented review of a study in Bangladesh, it was substantiated that the respondents experienced stress as 33.3% and 46.92% underwent a range of low to higher degrees of form of stress (Li et al. 2021). The study attributed an increased distress in mental state of individuals who spent over 4 h accessing information on the COVID-19 pandemic (Saravanan et al. 2020). Although humans need some degree of stress to perform optimally (Gustems-Carnicer et al. 2020), higher grades of mental stress in lecturers are directly reported to be an influencer of maladjustment to stress among learners (Stapleton et al. 2020). The level of psychological distress is dependent on the rate of watching and listening to reports and documentation on digital space about the COVID-19 pandemic (Salari et al. 2020). This is called Infodemic, which is when the adverse effect stories from the media space affect the victims’ psychological state of the prone individuals (Giallonardo et al. 2020); this occurrence is associated with the COVID-19 situation. The high proportion of psychological distress in this study was probably because of news of deaths on the media as academics were at home watching television and using smartphones to get updated on the pandemic. The period also took a toll on the psyche of academics as some were confused and thought there was no hope in sight. For example, there was retrenchment of academic staff, and there was no monthly salary for academic staff in a few institutions, but this was not the case in this study setting.

### Coping strategy

A significant finding showed that the most rated statement was ‘I try to lose myself for a while by drinking alcohol or taking drugs’ 3.8 ± 0.60. This is contrary to a study that reported 2% of the respondents indulging in the use of substances to cope with psychological stress as such practice may not be controlled after some period (Walke & Samuel 2018). The variance in the study was probably because the study was conducted before the COVID-19 pandemic. Avoidance strategy of coping could lead to the emergence of maiden mental distress, for instance, indulgence in substance use such as alcohol, tobacco, among others, could induce extra problems (Gustems-Carnicer et al. 2020). In a study cited in another literature, which is similar to the current study, it was reported that in the United States of America and Canada, 20% of the respondents resorted to the use of substances to cope (Walke & Samuel 2018). The present coping strategy used by the lecturers in this study which is an intake of alcohol or drugs is not in tandem with the reality to address psychological stress. However, frustration at that moment may be the factor that propelled the lecturer to use it as a coping strategy. The least rated statement was ‘I look for something good in what is happening’ 1.9 ± 1.16. The overall statements revealed a mean of 2.3. This implies that the respondents did not use much of the positive reinterpretation and growth as a coping style. This is incongruent with a study by Monfred (2021) where ‘listening to music and spending time on social media’ was reported to be the most documented psychological distress among the respondents. In a study by Li et al. (2021), it was revealed that the majority of the staff (91.77%) had a positive strategy for coping. Individuals who are curious to get an update on COVID-19 from the media have increased worries as the majority of the information available were worrisome coupled with a little of untrue reports, which may aggravate psychological distress in a person (Salari et al. 2020). Lecturers who put up an adaptive method to cope showed an improved mental health state and it mitigated the effect of inappropriate mood caused by protracted periods of stress (Stapleton et al. 2020). A study by Walke & Samuel in 2018 stated that 58% of the respondents engaged themselves with fantasy, going shopping and watching movies on screen. The same study reported such engagement as self-distraction, which could further exacerbate the features of psychological stress in an individual (Walke & Samuel 2018). The least used coping strategy in this study seems to corroborate the loss of hope that was associated with the pandemic among the academic staff. This could be partly because of media reports of death statistics, loss of loved ones, loss of job among, other factors. However, the use of alcohol reported may be to spark up the mood amid the present challenges occasioned by the COVID-19 pandemic.

### Coping strategies and psychological distress

The present study showed that substance abuse, behavioural disengagement, focus on and venting of emotions and positive reinterpretation and growth are predictors of psychological distress. In another similar study that utilised regression model carried out among Australia teachers, the study showed that active coping mechanism is a predictor of psychological distress among the teachers (Stapleton et al. 2020). A study in China, which was among both academics and medical students posited that coping strategy is linked to psychological stress, and there was a significant difference between coping and psychological stress among the respondents (Li et al. 2021). Substance use could negatively impact the brain thereby heightened psychological distress among the academics. The academics get upset as reported in this study, the coping strategy that is ‘focus on and venting emotions’ could also be induced by the intake of the substance as irritability could be associated with the use of substance. The net effect of such cyclical events could impact mood, thereby causing psychological distress among the academics.

### Recommendations

The study showed a high level of psychological distress among the academics. The coping strategy mostly used was substance use. The study thereby recommends teaching and training on adaptive coping styles among the staff of the institution. The school authority should also be prepared to provide social support to academics in the event of future probable pandemics.

### Limitations

The study was conducted during the COVID-19 pandemic. The study was limited to utilising questionnaires as a data collection tool among the respondents. The study would have adopted a mixed method using a phenomenological to capture the experiences. This would have enriched and further corroborated the present findings but for strict adherence to COVID-19 pandemic rules, an in-depth interview could not be undertaken.

## Conclusion

The study concluded that there was a high level of psychological distress among the respondents, which is an indication of severe stress. Most of the respondents also reported feeling worthless. The coping strategy utilised by the respondents was taking alcohol or drugs and few of the respondents used a positive reinterpretation and growth as a coping style as they looked for something good in what is happening. The awareness of an effective and adaptive coping strategy is imperative to safeguard the mental health of the academics.
